# Loss of ectonucleotidases from the coronary vascular bed after ischemia-reperfusion in isolated rat heart

**DOI:** 10.1186/1471-2261-13-53

**Published:** 2013-07-28

**Authors:** Kaoru Takahashi-Sato, Masahiro Murakawa, Junko Kimura, Masa-aki Ito, Isao Matsuoka

**Affiliations:** 1 Departments of Anesthesiology, Fukushima Medical University School of Medicine, Fukushima 960-1295, Japan; 2 Departments of Pharmacology, Fukushima Medical University School of Medicine, Fukushima 960-1295, Japan; 3 Laboratory of Pharmacology, Faculty of Pharmacy, Takasaki University of Health and Welfare, Gunma 370-0033, Japan

**Keywords:** Ischemia-reperfusion, Coronary circulation, Ectonucleotidase, ATP, Adenosine

## Abstract

**Background:**

Ectonucleotidase plays an important role in the regulation of cardiac function by controlling extracellular levels of adenine nucleotides and adenosine. To determine the influence of ischemia-reperfusion injury on ectonucleotidase activity in coronary vascular bed, we compared the metabolic profile of adenine nucleotides during the coronary circulation in pre- and post-ischemic heart.

**Methods:**

Langendorff-perfused rat hearts were used to assess the intracoronary metabolism of adenine nucleotides. The effects of ischemia on the adenine nucleotide metabolism were examined after 30 min of ischemia and 30 min of reperfusion. Adenine nucleotide metabolites were measured by high performance liquid chromatography.

**Results:**

ATP, ADP and AMP were rapidly metabolized to adenosine and inosine during the coronary circulation. After ischemia, ectonucleotidase activity of the coronary vascular bed was significantly decreased. In addition, the perfusate from the ischemic heart contained a considerable amount of enzymes degrading ATP, AMP and adenosine. Immunoblot analysis revealed that the perfusate from the ischemic heart dominantly contained ectonucleoside triphosphate diphosphohydrolase 1, and, to a lesser extent, ecto-5’-nucleotidase. The leakage of nucleotide metabolizing enzymes from the coronary vascular bed by ischemia-reperfusion was more remarkable in aged rats, in which post-ischemic cardiac dysfunction was more serious.

**Conclusion:**

Ectonucleotidases were liberated from the coronary vascular bed by ischemia-reperfusion, resulting in an overall decrease in ectonucleotidase activity in the post-ischemic coronary vascular bed. These results suggest that decreased ectonucleotidase activity by ischemia may exacerbate subsequent reperfusion injury, and that levels of circulating ectonucleotidase may reflect the severity of ischemic vascular injury.

## Background

Adenine nucleotides and adenosine (Ado) are endogenous modulators of cardiac function. Ado has cardiodepressive effects, such as the negative chronotropic effect and the inhibition of inotropic action of β adrenergic agonists, via activation of the Ado A_1_ receptor [1]. In contrast to Ado, ATP itself has positive inotropic and vasoconstricting actions via the P2X ionotropic receptors [2]. However, intravenous ATP has effects similar to those of Ado through its rapid degradation to Ado [2-4]. Several lines of evidence indicate that ATP is released from a wide variety of cell types, such as endothelial cells, vascular smooth muscle cells and platelets by physiological and mechanical stimuli [5]. Therefore, regulated conversion of extracellular ATP to Ado plays an important role in purinergic regulation of cardiac function.

Extracellular ATP catabolism is mediated by several ectoenzymes, such as ectonucleoside triphosphate diphosphohydrolases (ENTPD), ectonucleotide pyrophosphatases/phosphodiesterases and ecto-5’-nucleotidase (CD73) [6]. In the coronary vascular bed, ENTPD1 (CD39) [7,8] and CD73 [9] are thought to be involved in the conversion of ATP to Ado. Recent studies suggested that ectonucleotidase activity is altered under pathophysiological conditions of the heart, such as myocardial ischemia and chronic heart failure [10-13]. Activation of CD73 was found in the preconditioned heart, which was induced by brief periods of myocardial ischemia [11]. In contrast, oxidative stress and inflammatory cytokines inactivate CD39 on the luminal surface of blood vessels, which in turn lead to increased platelet aggregation [12]. These observations suggest that individual enzymes involved in ATP catabolism may be affected differently under various pathophysiological conditions, such as ischemia-reperfusion injury.

In the present study, we examined ectonucleotidase activity in the coronary vascular bed by administrating adenine nucleotide substrates into the coronary circulation of isolated rat hearts, and the effects of ischemia-reperfusion on intracoronary ATP catabolism were investigated.

## Methods

### Materials

ATP, ADP, AMP, Ado, α,β-methylene adenosine diphosphate (α,β-MeADP), hypoxanthine, inosine, levamisole, ouabain, diethylpyrocarbonate were obtained from Sigma-Aldrich (St. Louis, MO, USA). 1,N^6^-etheno adenosine-5’-triphosphate (eATP), 1,N^6^-etheno adenosine-5’-diphosphate (eADP), 1,N^6^-etheno adenosine-5’-monophosphate (eAMP) and 1,N^6^-etheno adenosine (eAdo) were obtained from MP Biomedicals (Solon, OH, USA). ARL67156 was from Tocris Bioscience (Ellisville, MO, USA). Anti-rat CD39 polyclonal guinea pig antiserum and anti-rat CD73 monoclonal mouse antibody were obtained from Neuromics (Bloomington, MN, USA) and BD Biosciences (San Jose, CA, USA), respectively. All other reagents were of the highest purity available.

### Experimental animals

All animal experiments were performed in accordance with the regulations of the Animal Research Committee of Fukushima Medical University School of Medicine. In this study, totally forty five male Wistar rats were used for experiments; fifteen 8–10 weeks old rats for analysis of the adenine nucleotide metabolism in the normal coronary circulation, ten 8–10 weeks old rats (5 control and 5 ischemia-reperfusion) for examining the effects of ischemia reperfusion on the adenine nucleotide metabolism in the coronary circulation, and ten aged rats (24 months old, 5 control and 5 ischemia-reperfusion) and ten young adult rats (8 weeks old, 5 control and 5 ischemia-reperfusion) for evaluating the effects of aging.

### Isolated heart perfusion

Rats were anesthetized by intra-peritoneal injections of 40 mg/kg sodium pentobarbital with 1000 U/kg heparin. Under conditions of artificial ventilation, the heart was rapidly excised and immediately mounted on a Langendorff apparatus, and perfused with a physiological salt (PS) solution at a constant flow rate of 5–8 mL/min under which perfusion pressure was maintained 60–70 mmHg, and was allowed to beat at an intrinsic heart rate without pacing throughout the experiments. The PS solution contained (in mmol/L): NaCl 130, NaHCO_3_ 14.9, KCl 4.7, MgSO_4_ 1.2, KH_2_PO_4_ 1.2, CaCl_2_ 1.8 and glucose 5.5. It was filtered through a nylon filter (0.4 μm, Corning, NY, USA) to remove contaminants, and gassed continuously with 95% O_2_ and 5% CO_2_, which resulted in a pH of 7.35-7.4 and a pO_2_ > 80 kPa. The heart was housed in a chamber that was maintained at 37°C. A pressure transducer (TP-200 T, Nihon Kohden, Tokyo, Japan) was connected to the perfusion line, and perfusion pressure and heart rate (HR, beat /min) were monitored through a carrier amplifier (AP601G, Nihon Kohden, Tokyo, Japan). Data were continuously recorded and analyzed using MacLab (AD-Instruments Pty Ltd., Castle Hill, Australia). Normally, the heart rate ranged from 200 to 250 beats/min after 20 min of control perfusion.

### Measurement of ectonucleotidase activity in the coronary vascular bed

After stabilization of the beating heart for 20 min, PS solution (0.3 ml) containing ATP, ADP, AMP and Ado at 100 μM was administered using an injection loop connected to a perfusion line by three-way stopcocks at 5 min intervals. With this system, injection did not affect the perfusion pressure. Test substrates were introduced into the coronary circulation 20 sec after the injection, and most of the substrates and their metabolites were eluted from the heart within 1 min after the injection. Samples of coronary effluent from the heart were collected once for 20 sec immediately before, and five times every 20 sec after injection of substrates. Aliquots of effluent (0.1 ml) were immediately mixed with an equal volume of 10 mM ethylenediaminetetraacetic acid (EDTA) and heated at 80°C for 5 min. This treatment prevented the metabolites from further degradation by endogenous nucleotidases released from the heart. The first minute fraction after injection of the substrates were pooled and adenine nucleotide metabolites were analyzed using high performance liquid chromatography (HPLC) with a UV detector as described below. In preliminary experiments, we confirmed that the metabolic profile of each substrate during coronary circulation was not altered by repeated application at least 5 times. In another set of experiments, eATP, eADP, eAMP and eAdo at 10 μM were also used as substrates. These 1,N^6^-etheno derivatives are good substrates for ectonucleotidases [14], and their metabolites could be monitored with a fluorescent detector, separated from endogenous adenine nucleotides released during coronary circulation. The etheno derivatives were administrated and analyzed with the same protocol described for adenine compound substrates.

### Effects of global ischemia on ectonucleotidase activity

After a 20 min stabilization of the heart, global ischemia was induced by stopping the coronary perfusion by changing to the surface perfusion using a three-way stopcocks located above the aortic cannula. Therefore, heart was not dry and its temperature was maintained at 37°C during the global ischemia. After 30 min of ischemia, the heart was reperfused with normal PS solution for 30 min, and ectonucleotidase activity in the coronary vascular bed was measured. In the experiments with the post-ischemic heart, eATP and eAMP at 10 μM were used as substrates for ectonucleotidase. Since eAdo was not metabolized by or taken up into the coronary vascular bed, Ado (100 μM) was used to determine the change in adenosine deaminase (ADA) activity. At 5 min intervals 0.3 ml of each substrate was injected. Collection of effluents and analyses of metabolites were performed as described above. The control heart was perfused with normal PS solution for 80 min without ischemia and then the ectonucleotidase activity was examined in the same way as that for the ischemic heart.

### Ischemia-induced release of adenine nucleotides and ectonucleotidase

Ischemia was induced as described above. Immediately after starting reperfusion, effluents were collected for 2 min at 20 sec intervals. Effluents before ischemia and 30 min after reperfusion were also collected to measure the adenine nucleotide levels in the normal and post-ischemia recovery states, respectively. Aliquots of 200 μl of samples were mixed immediately with 200 μl of 10 mM EDTA and heated at 80°C for 5 min. The levels of adenine nucleotides, Ado, inosine and hypoxanthine were measured by HPLC. Ectonucleotidase activity in the effluent was investigated by adding 0.2 ml of 10?μM eATP, eAMP, or 100 μM Ado to 0.2 ml of the effluents. After 10 min of incubation at room temperature, 0.4 ml of 10 mM EDTA was added and the samples were heated at 80°C for 5 min to stop the reaction. The metabolites were analyzed by HPLC. The concentrations of substrates remaining were corrected by subtracting the concentrations of endogenous compounds present in the samples, such as ATP and Ado, after parallel incubation without added substrates.

### Measurement of LDH release from ischemic heart

LDH activity in the first minute coronary effluents from ischemic heart was measured according to manufacturer instructions, using Cytotoxicity Detection Kit (Roche Applied Science, Indianapolis, IN).

### HPLC analysis of adenine nucleotide metabolites

The samples containing adenine nucleotide metabolites were analyzed using a JASCO HPLC system equipped with an analytical YMC-Pack ODS-A column (S-5, 4.6 X 100 mm, YMC Inc. Kyoto, Japan) equilibrated at 40°C with 50 mM NaH_2_PO_4_ (pH 5.5 adjusted with H_3_PO_4_) at a flow rate of 1 ml/min [15,16]. The HPLC system consisted of a DG-980-50 degasser, LG-980-20 ternary gradient unit, PO-980 pump, AS-950 autoinjector, CO-966 column oven, MD-915 multi-wavelength detector and FP-920 fluorescence detector (all from JASCO Corporation, Tokyo, Japan). Samples were filtered through 0.2 mm nylon filters and kept at 4°C. The injection of samples was followed by a 3 min flow of 50 mM NaH_2_ PO_4_ (pH 5.5) and a 5 min flow of a linear gradient of 0 -20% (vol/ vol) methanol in 50 mM NaH_2_PO_4_ (pH 5.5). After a 5 min flow of 20% methanol in 50 mM NaH_2_PO_4_ (pH 5.5), the eluent was changed to 50 mM NaH_2_PO_4_ (pH 5.5) and elution was continued for 7 min before the next sample was injected. The range of wavelengths scanned was 190–500 nm, and the absorption maxima used for determining ATP, ADP, AMP and Ado were 260 nm and for inosine 247 nm and hypoxanthine 252 nm. The eATP metabolites were determined fluorometrically with excitation and emission wavelengths at 270 and 410 nm, respectively. The peaks were identified by comparison with the retention times of standards.

### Dot blot analysis

The existences of CD39 and CD73 in the effluents from the ischemic heart were examined by dot blot analysis. The effluents from pre- and post-ischemic heat were applied to nitrocellulose membrane at the volume of 500 μl by using a vacuum blotting system. Cell lysates (10 μg /100 μl) obtained from HEK293 cells transfected with a pcDNA3.1 expression vector containing full length cDNA of rat CD39 , rat CD73 or green fluorescent protein were used as positive and negative controls. The membrane was then blocked with 3% BSA in Tris- buffered saline containing 0.1% Tween-20 (TBST, pH 7.4), followed by incubating anti-rat CD39 antiserum or anti-rat CD73 monoclonal antibody diluted (1:500) with TBST containing 1% BAS. The membranes were washed three times with TBST and then incubated with horseradish peroxidase conjugated second antibody. The signals were detected by ECL-plus using LAS-3000 imaging system (Fuji Photo Film Co. Tokyo, Japan).

### Statistical analysis

Results are presented as the means ± S.E.M. Statistical analyses of the data were performed by the unpaired Student’s *t*-test for two data comparison and one-way analysis of variance (ANOVA) with the Dunnett two tailed test for multiple data comparison. Pearson correlation coefficients were calculated to examine the relationship between the decrease in vascular ATPase activity and the leakage of ATPase into coronary effluent after ischemia-reperfusion. *P* values less than 0.05 were considered to be significant.

## Results

### Effects of adenine nucleotides and Ado on cardiac function

We examined the effects of the administration of adenine nucleotides and Ado into the coronary circulation on the cardiac function using the Langendorff perfusion of isolated rat hearts. Addition of ATP, ADP, AMP or Ado at 200 μM to the perfusate caused a sudden bradycardia with decreased perfusion pressure. A few seconds after stopping, the heart started to beat again with a sinus rhythm, and it took about 1 min to recover the heart rate and perfusion pressure. All the nucleotides and Ado had similar effects (data not shown). Therefore, in the following experiments, we assessed how the nucleotides were metabolized in the coronary circulation.

### Metabolisms of adenine nucleotides and Ado in coronary vascular bed

**Figure 1:**
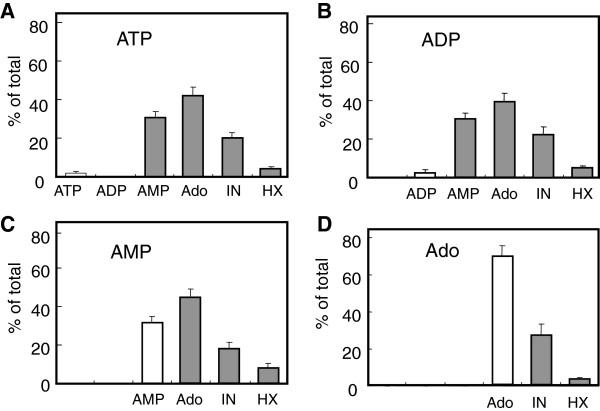
**Metabolism of ATP (A), ADP (B), AMP (C) and adenosine (Ado, D) during a single pass of coronary circulation.** Each substrate at 200 μM (0.3 ml) was added to the coronary perfusate after 20 min stabilization of the normal hearts. Metabolites in the coronary effluents of the first one minute fraction after injection of the substrate were measured by HPLC (*Open columns;* initial substrate, *black columns;* metabolites). Data are shown as percent of total metabolites collected. All values are means ± S.E.M (n = 13).

The coronary effluent from the Langendorff-perfused heart did not contain any detectable adenine nucleotides or their metabolites. When ATP and ADP were injected into the perfusate, they were almost completely metabolized to AMP and Ado during a single pass (5–10 sec) through the coronary circulation (Figure 1A, B). A considerable amount of Ado converted from ATP or ADP was further metabolized to inosine and hypoxanthine. When AMP was used as a substrate, nearly 70% of it was converted to Ado, which was also further metabolized to inosine and hypoxanthine (Figure 1C). The relative amounts of metabolites, e.g. AMP (30%), Ado (40-45%), inosine (20%) and hypoxanthine (5-10%) in the collected effluents, were similar regardless of whether ATP, ADP or AMP was used as a substrate, suggesting that enzyme activities hydrolyzing ATP and ADP were extremely high in the coronary vascular bed. In contrast, only about 30% of Ado injected was converted to inosine (Figure 1D), suggesting that Ado metabolism was relatively slow. Total recoveries of injected ATP, ADP, AMP and Ado were 50–60%.

**Figure 2:**
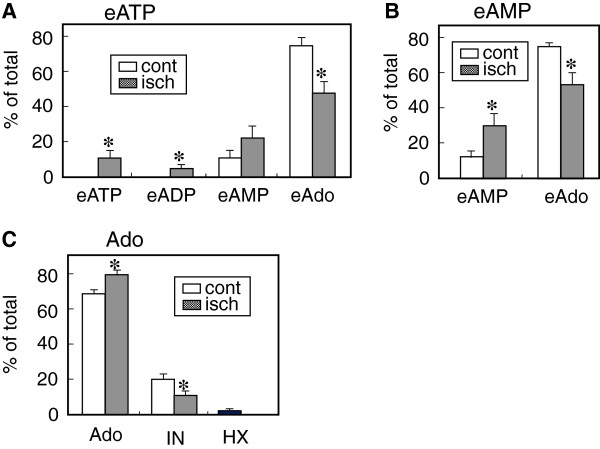
**Effect of ischemia and reperfusion on metabolism of eATP (A), eAMP (B) and adenosine (C).** After 20 min equilibration, the hearts (n = 5) were subjected to a 30 min-ischemia, followed by reperfusion for 30 min. Each substrate at 10 μM (0.3 ml) was added to the coronary perfusate and subsequent metabolites in the coronary effluents of the first one minute fraction after injection of the substrate were measured by HPLC. Control metabolism was examined in heart after a time-matched control perfusion (80 min, n = 5). *Open columns;* metabolites from the control hearts, *dark columns;* metabolites from ischemic heart. Data are shown as percent of total metabolites collected. All values are means ± S.E.M (n = 5). *Significantly different from the control value at P < 0.05 by Student’s *t*-test.

Similar metabolic profiles were obtained with etheno-derivatives of adenine nucleotides except for eAdo, which seemed to be the final metabolite (data not shown, see Figure 2). Furthermore, the total recoveries of injected etheno-substrates, including eAdo, were more than 90%, suggesting that eAdo may not be uptake during coronary circulation.

### Effect of ischemia on ectonucleotidase activity in the coronary vascular bed

**Figure 3:**
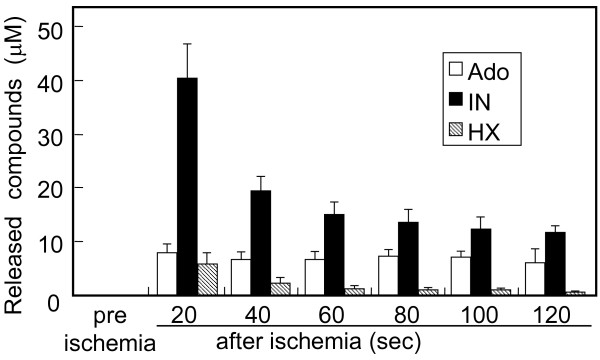
**Adenine nucleotide metabolites in the effluents immediately after reperfusion.** After 30 min-ischemia, hearts were reperfused with a normal solution, and effluents were collected every 20 sec for 120 sec. Effluents were also collected at pre-ischemia. Endogenous adenine nucleotide metabolites released from hearts into the effluents were measured by HPLC. Data are means ±S.E.M (n = 10). Ado; adenosine, IN; inosine, HX; hypoxanthine.

When ischemia was induced by stopping the perfusion inflow to the heart, spontaneous beating was arrested within 10 min. After 30 min of ischemia, reperfusion with normal PS solution resulted in a transient increase in perfusion pressure (125-140% of pre-ischemia). The increased pressure was decreased to the pre-ischemic levels within 30 sec. Spontaneous heart beating was restored within 30 seconds at the latest with a decreased HR (60–75% of pre-ischemia), accompanied by serious arrhythmia. The first 20 sec-fraction of reperfusate (~1.5 ml) from ischemic heart contained Ado (8.0 ± 1.5 μM, n = 10) and inosine (40.4 ± 6.6 μM, n = 10), which were absent in the perfusate from pre-ischemic heart (Figure 3). The high level of inosine in the perfusate was gradually decreased, whereas the Ado level was maintained for 2 min following the start of reperfusion (Figure 3).

We next examined the ectonucleotidase activities in the coronary vascular bed of the post-ischemic heart using eATP, eAMP as substrates (Figure 2). In the control heart, eATP (10 μM) was completely hydrolyzed to eAMP, which was further partly metabolized to eAdo (Figure 2A). The conversion of eAMP to eAdo was greater compared to that of the natural substrate AMP (Figure 1C and 2B). This may be due to a lack of reuptake of eAdo, resulting in eAdo accumulation. The metabolism of eATP and eAMP in the ischemic heart was clearly impaired (Figure 2A, B). Since eAdo is not a substrate for ADA, Ado itself was used as substrate to examine the change in Ado metabolism in the post-ischemic heart. As shown in Figure 2C, the conversion of Ado to inosine was also decreased in the ischemic heart.

### Ectonucleotidase activity in the effluent from reperfusion heart

**Figure 4:**
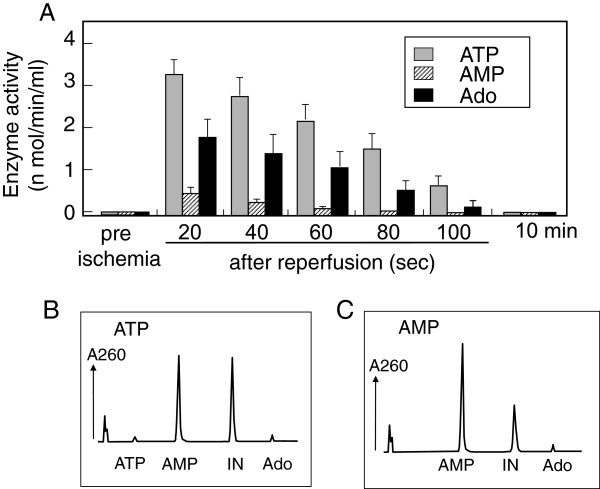
**Ectonucleotidase activity in the effluents of immediately after reperfusion. (A)** Effluents collected as indicated in Figure 3 were incubated with 100 μM ATP, AMP or adenosine (Ado) for 10 or 30 min, and the decrease in each substrate was measured by HPLC. All values are means ± S.E.M (n = 5)Enzyme activities are shown as nmole substrate hydrolyzed by 1 ml effluent for 1 min. All values are means ± S.E.M (n = 5). Lower panels show typical HPLC chromatograms of ATP **(B)** and AMP **(C)** hydrolyzed by the first fraction of post-ischemic reperfusate.

**Figure 5:**
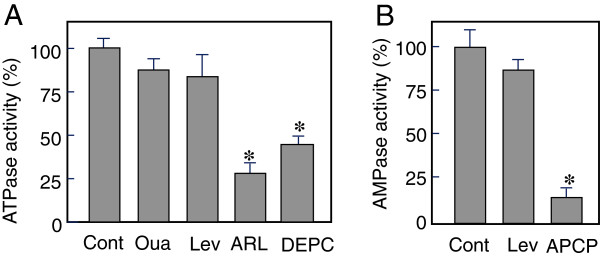
**Characteristics of ATPase and AMPase activity in the effluents of immediately after reperfusion. A**: The first and second fractions (totally 40 sec after reperfusion) were pooled. Aliquots of 100 μl sample were preincubated for 5 min with saline (control), ouabain (Oua, 10 μM), levamisole (Lev, 500 μM), diethylpyrocarbonate (DEPC, 500 μM), ARL67156 (ARL, 50 μM) or α,β-MeADP **(**APCP, 10 μM, only in **B)**, followed by adding ATP **(**100 μM, **A)** or AMP **(**100 μM, **B)**. The reactions were performed at 37°C for 5 min in A or 30 min in **B**, and the substrates remained were measured by HPLC. Data shown are percentage of initial substrate levels. Values are means ± S.E.M (n = 5). *Significantly different from the control value at P < 0.05 by ANOVA, post-hoc Dunnett analysis.

**Figure 6:**
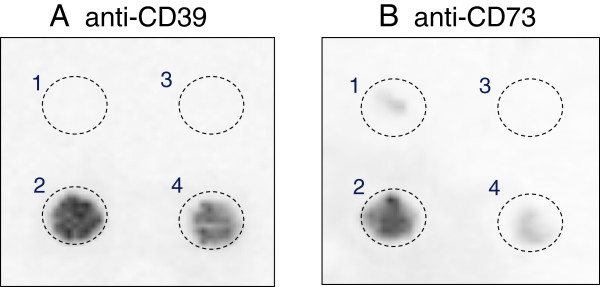
**Detection of immunoreactive CD39 and CD73 in the effluent of ischemia reperfusion.** The effluent samples (300 μl) from pre-ischemia (3) and ischemia-reperfusion hearts (4) were applied to nitrocellulose membrane, and subjected to dot blot analysis with anti-CD39 antibody **(A)** and anti-CD73 antibody **(B)**. As the negative and positive control, membrane extract (30 μg protein in 300 μl) from HEK293 cells transfected with control pcDNA3vector **(**1 in **A** and **B)** or CD39- **(**2 in **A)** and CD73-expressing plasmid **(**2 in **B)** were used. Results shown are representative of three separate sets of experiments.

During the measurements of endogenously released metabolites in the post-ischemic reperfusates, we found that the relative amounts of AMP, Ado and inosine in the reperfusates were changed considerably unless the samples were not heated at 80°C. This suggested that the post-ischemic reperfusate contained enzyme(s) which catalyze nucleotide metabolism. As shown in Figure 4A, the perfusates before ischemia did not contain any nucleotidase activity. However, perfusates after ischemia contained enzymes that hydrolyzed ATP, AMP and Ado. The ATPase activity was most remarkable, and Ado degradation, which was due to ADA because inosine, detected as a product, was also prominent, as compared with AMPase activity (Figure 4A). These enzyme activities were highest in the first 20-sec fraction of reperfusate, and then gradually decreased. After 30 min reperfusion, no enzyme activity was detected in the effluent. Figure 4B and C show typical HPLC chromatograms, after hydrolyzing ATP and AMP, respectively. ATP was hydrolyzed directly to AMP without generating ADP, and Ado generated from AMP was largely converted to inosine. ATP hydrolysis was markedly inhibited by NTPDase inhibitors, ARL67156 (50 μM) and diethylpyrocarbonate (500 μM), but not by ouabain (10 μM), a Na^+^-K^+^-ATPase inhibitor or levamisole (500 μM), an alkaline phosphatase inhibitor (Figure 5). In addition, the CD73 inhibitor α,β-MeADP (10 μM) abolished AMP hydrolysis (Figure 5). Actually, the presence of immunoreactive CD39 or CD73 was detected in the first 20-sec fraction of reperfusate by dot blot analysis (Figure 6), although CD73 level was very small.

### Effects of aging on ectonucleotidase leakage induced by ischemia reperfusion

**Figure 7:**
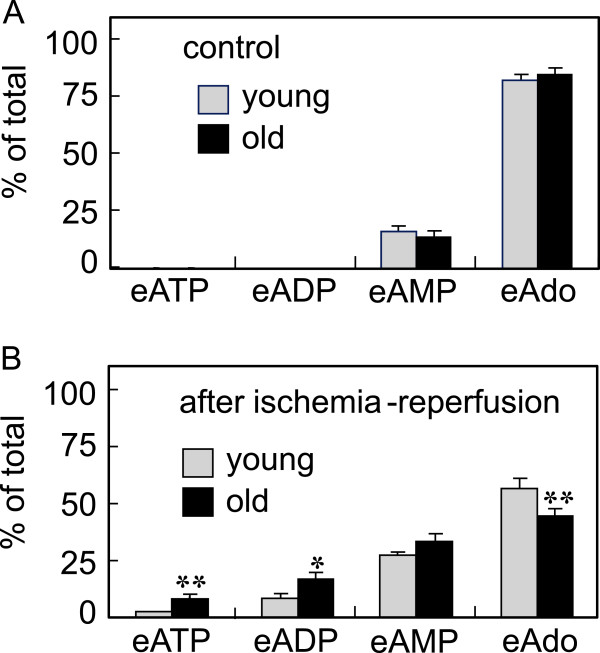
**Effects of aging on the changes in ATP hydrolysis after ischemia-reperfusion in the coronary circulation .** Control perfusion and ischemia-reperfusion were performed as indicated in Figure 2 in young adult and aged rats. A substrate of eATP (10 μM, 0.3 ml) was administrated into the coronary perfusate and subsequent metabolites in the effluent were measured by HPLC. Data are shown as percent of total metabolites collected from control hearts **(A)** and ischemia-reperfusion hearts **(B)**. Gray columns: young adult rats , black columns: aged rats. All values are means ± S.E.M (n = 5). *P < 0.05 , **P < 0.01 significantly different from the control value by Student’s *t*-test.

**Figure 8:**
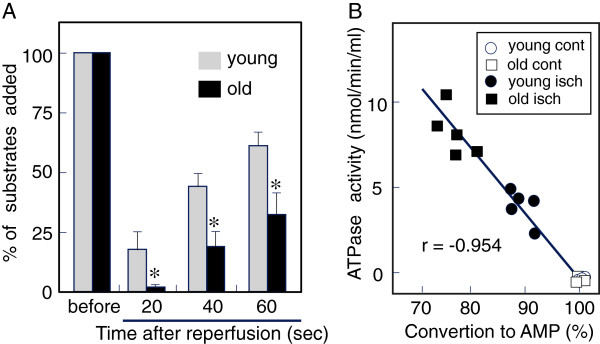
**Effects of aging on the leakage of ATPase from coronary vascular bed by ischemia-reperfusion.** In the ischemia-reperfusion experiments shown in Figure 7, the effluents immediately after reperfusion (20 second-fraction for 1 min) were collected and ATPase activity in each sample was determined by incubating with ATP (100 μM) for 5 min **(A)**. Data shown are levels of substrate ATP that remained in the reaction mixture of young adult rats (gray column) and aged rats (black column). Values are means ± S.E.M (n = 5). *Significantly different from the control value at *P < 0.05 by Student’s *t*-test. **(B)**: A correlation analysis was performed on the results presented in Figure 7 and Figure 8A from both young adult and aged rats (n = 20).

It is well known that aging is a major risk factor for the cardiovascular disorder induced by ischemia. We therefore examined whether ischemia-induced loss of ecto-nucleotidase from the coronary vascular bed was changed by aging. ATP hydrolysis activity in pre-ischemic heart in aged rats (24 month old) was not different from 8-week old young adult rats (Figure 7A). However, ischemia-induced decrease in ATP hydrolysis was more significant in aged rats as compared to young rats (Figure 7B). Furthermore, the content of ATPase activity in the effuluent from ischemic heart was much higher in aged rats (Figure 8A). When data obtained from the control and ischemic heart from both young and aged rats were summarized on a scatter plot, there was a strong negative correlation between the ATPase activity leaked in the reperfusate and the decrease in ATPase activity of coronary vascular bed (r = − 0.978, P < 0.0001, Figure 8B). With respect to functional responses, spontaneous heart beating was restored within 10 seconds of reperfusion after 30 min-ischemia in all young adult 8-week-old rats, whereas functional recovery was observed in only 2 out of 5 aged rats. In addition, LDH release from ischemic heart of aged rat (356 ± 18 mU/ml, n = 4) was significantly higher (p < 0.05) than those of young rat (215 + 32 mU/ml, n =5).

## Discussion

Although it is well known that the vascular endothelial cells possess highly active enzymes that hydrolyze extracellular nucleotides [7-9], only limited information about the overall metabolic profile of adenine nucleotides in the coronary circulation is available in literature [3]. The present study showed that the coronary vascular bed possesses highly active ectoenzymes which catalyze ATP hydrolysis. After a single pass of the coronary circulation, ATP and ADP were converted almost totally to AMP, and nearly 70% of the AMP was further metabolized to Ado and inosine. The relative amounts of the final metabolites from three different substrates, ATP, ADP and AMP, were very similar; 30% AMP, 40-45% Ado, 20% inosine and 5-10% hypoxanthine of the total metabolites collected. This indicates that ATPase and ADPase activities are much higher than AMPase activity, and that the conversion of AMP to Ado is the rate-limiting step in the formation of Ado from adenine nucleotides in the rat heart coronary circulation. The levels of inosine, which is produced from Ado by ADA, were also similar when ATP, ADP, AMP or Ado were used as substrates, showing that 30-40% of Ado was converted to inosine. Thus, the relative potencies of ectonucleotidase activities in the rat coronary vascular bed were ATPase, ADPase > AMPase > ADA. Since coronary endothelium expresses CD39 and CD73, it is suggested that ATP/ADPase and AMPase activities are due to CD39 and CD73, respectively. These results are consistent with earlier work reported by Korchazhkina et al. [3].

The present results also demonstrated that ectonucleotidase activity in the coronary vascular bed was significantly decreased by ischemia-reperfusion. During the acute phase of reperfusion, Ado and its metabolites, inosine and hypoxanthine, were released into the perfusate. To distinguish between endogenous compounds and metabolites of exogenous substrates, we used eATP and eAMP to assess ectonucleotidase activity in the pre- and post-ischemic coronary circulation. The eATP and eAMP were metabolized in a manner similar to ATP and AMP. However, eAdo was the final metabolite from eATP or eAMP, because it did not seem to be the substrate for ADA and Ado transporter. In the control heart, eATP was completely converted to eAMP, which metabolized further to eAdo during the coronary circulation. With this system, we clearly showed that the hydrolysis of eATP and eAMP was significantly decreased after 30 min ischemia. Although it has been shown that oxidative stress and proinflammatory cytokines inhibit CD39 activity in the endothelial cells [12] and the renal vascular bed [17], little is known about the direct effect of ischemia-reperfusion on CD39 activity in the coronary vascular bed. Several studies have demonstrated that up-regulation of CD39 and CD73 is involved in the cardioprotection by ischemia preconditioning. Since preconditioning is induced by repetitive short period ischemia (generally for 5 min each), it reflects an adaptive phenomenon rather than acute change after prolonged ischemia. Indeed, significant up-regulation of CD39 and CD73 activities have been reported in post-ischemic brains several days later, which seems to be also an adaptation to post-ischemic inflammation [18,19]. The present results show an acute change in ectonucleotidase activities after ischemia.

Several mechanisms have been proposed for the alteration of ectonucleotidase activity by pathophysiological stimuli. With respect to the impairment of ectonucleotidase activity, oxidative inactivation of CD39and CD73 was suggested from observations with NO generating agents [20], inflammatory cytokine [21] and bacterial lipopolysaccharide [22]. In the present study, we found that different ectonucleotidases were liberated from the coronary vascular bed of the ischemic heart. Since cardiac tissue contains a relatively high level of cytosolic 5’-nucleotidase [23], as well as various ATP-dependent enzymes, ATPase and AMPase activities in the post-ischemic reperfusate may reflect the leakage of cytosolic enzymes from necrotic cells. However, the following lines of evidence suggest that the nucleotidase activities detected in the post-ischemic reperfusate are originated mainly from ectoenzymes. First, HPLC analysis clearly showed that the ATP hydrolyzing enzymes in the reperfusate had NTPDase activity, since ATP was directly converted to AMP without generating ADP, being consistent with the characteristic of CD39-meditated ATP hydrolysis. If intracellular ATPase is involved in ATP hydrolysis, the metabolites should include ADP. Second, ATP hydrolysis was inhibited by the CD39 inhibitors ARL67156 and diethylpyrocarbonate, but not by the intracellular ATPase inhibitors ouabain. Third, AMP hydrolysis was inhibited by α,β-MeADP, which selectively inhibits CD73 but not cytosolic 5’-nucleotidase. Finally, dot bot analysis demonstrated that coronary effluents from the ischemic heart contained immune reactive CD39 and CD73. Therefore, we propose that the liberations of these enzymes are from the luminal surface of the coronary vascular bed.

It is well known that ischemic heart disease worsen with age [24,25]. In this study, we showed that decrease in ATP hydrolysis in coronary circulation by ischemia-reperfusion were more remarkable in aged rats. We also showed that ischemia-induced loss of ectonucleotidase from the coronary vascular bed was accompanied by an increase in ATPase release. There was a good negative correlation between change in ATP hydrolysis activity in coronary circulation and the degree of ATPase liberation from the ischemic heart. These results indicate that decrease in ATP hydrolysis in ischemic heart is due to the loss of ATPase activity from the luminal surface side of the coronary vascular bed. Since these changes were remarkable in aged rats, it is interesting to evaluate whether circulating ATPase level could be a marker of the ischemia-reperfusion injury in human.

The present study provides a novel mechanism that explains the decrease in ectonucleotidase activity in ischemic heart coronary vascular bed. Although the post-ischemic reperfusate contained different enzymes that hydrolyze ATP, AMP and adenosine, the ATPase activity, which may be due to CD39, was more remarkable compared to AMPase and ADA. The selective decrease in CD39 would increase the local concentrations of ATP and ADP, which may cause increased platelet aggregation and exacerbation of ischemia and reperfusion injury [12,13,17]. Therefore, it is important to clarify the mechanism underlying the selective CD39 decreased by ischemia-reperfusion.

Limitation of this study is that we showed the decrease in ectonucleotidase activity in coronary circulation after global ischemia, whereas the myocardial infarction in human heart is only subject to regional ischemia. Recently, Bönner et al. [26] reported a significant decrease in CD39 expression in coronary endothelial cells using *in vivo* mouse myocardial infarction model. It is interesting to examine whether similar mechanism shown in this study is involved in the down-regulation of CD39 expression in post-ischemic coronary endothelial cells in such *in vivo* model. Finally, we observed in preliminary experiments that administration of adenine nucleotides into the coronary circulation before induction of ischemia to evaluate the control ectonucleotidase activity resulted in attenuating the ischemia-reperfusion-induced loss of ectonucleotidase activity, so that we stopped to compare the ectonucleotidase activity in pre- and post-ischemic condition in the same heart, and designed protocol as shown in Figure 2. This may reflect the preconditioning effect of adenine nucleotides, especially adenosine, on ischemic-reperfusion injury as previously reported [11]. This may be important phenomenon that should be examined.

## Conclusion

In summary, this study demonstrates that the ischemia-reperfusion elicits an overall decrease in ecto-nucleotidase activities, including ATPDase, AMPase and ADA, in the coronary vascular bed. In addition, this study shows that these changes in ATP catabolism in coronary circulation might be due to a significant liberation of ectonucleotidases from ischemic coronary vascular bed. This idea was further supported in results obtained in aged rats by showing that there was good correlation between ischemia-reperfusion induced nucleotidase liberation and decrease in ATP hydrolysis activity in ischemic coronary circulation. These results may suggest that protection of ectonucleotidase liberation from the coronary vascular bed is important for maintaining the post-ischemic cardiac function. Further study is needed to explore the mechanism underlying ectonucleotidase liberation caused by ischemia.

## Abbreviations

Ado: Adenosine; ADA: Adenosine deaminase; CD73: Ecto-5’-nucleotidase; CD39: Ectonucleoside triphosphate diphosphohydrolases 1; EDTA: Ethylenediaminetetraacetic acid; eATP: 1,N^6^-etheno adenosine-5’-triphosphate; eADP: 1,N^6^-etheno adenosine-5’-diphosphate; eAMP: 1,N^6^-etheno adenosine-5’-monophosphate; eAdo: 1,N^6^-etheno adenosine; ENTPD: Ectonucleoside triphosphate diphosphohydrolases; HPLC: High performance liquid chromatography; α,β-MeADP: α,β-methylene adenosine diphosphate.

## Competing interests

The authors declare that they have no competing interests in relation to this manuscript.

## Author’s contributions

KTS carried out all experimental work and correction of drafted manuscript. MI, MM and JK coordinated the work, analyzed and interpreted data. IM designed the study, analyzed and interpreted the data, searched literature, and drafted the manuscript. All authors read and approved the final manuscript.

## Pre-publication history

The pre-publication history for this paper can be accessed here:

http://www.biomedcentral.com/1471-2261/13/53/prepub
